# Supplementary material for: A Systematic Review of MicroRNA Signatures Associated with the Progression of Leukoplakia with and without Epithelial Dysplasia
Source: Biomolecules. 2021 Dec 14;11(12):1879. doi: 10.3390/biom11121879 (PMC8699326; doi:10.3390/biom11121879)
Supplement: Supplementary file 1 [file biomolecules-11-01879-s001.zip › biomolecules-1500919-supplementary.pdf]

Supplementary tables/figures:

Figure S1: Illustration of the steps involved in selection of eligible studies for this systematic

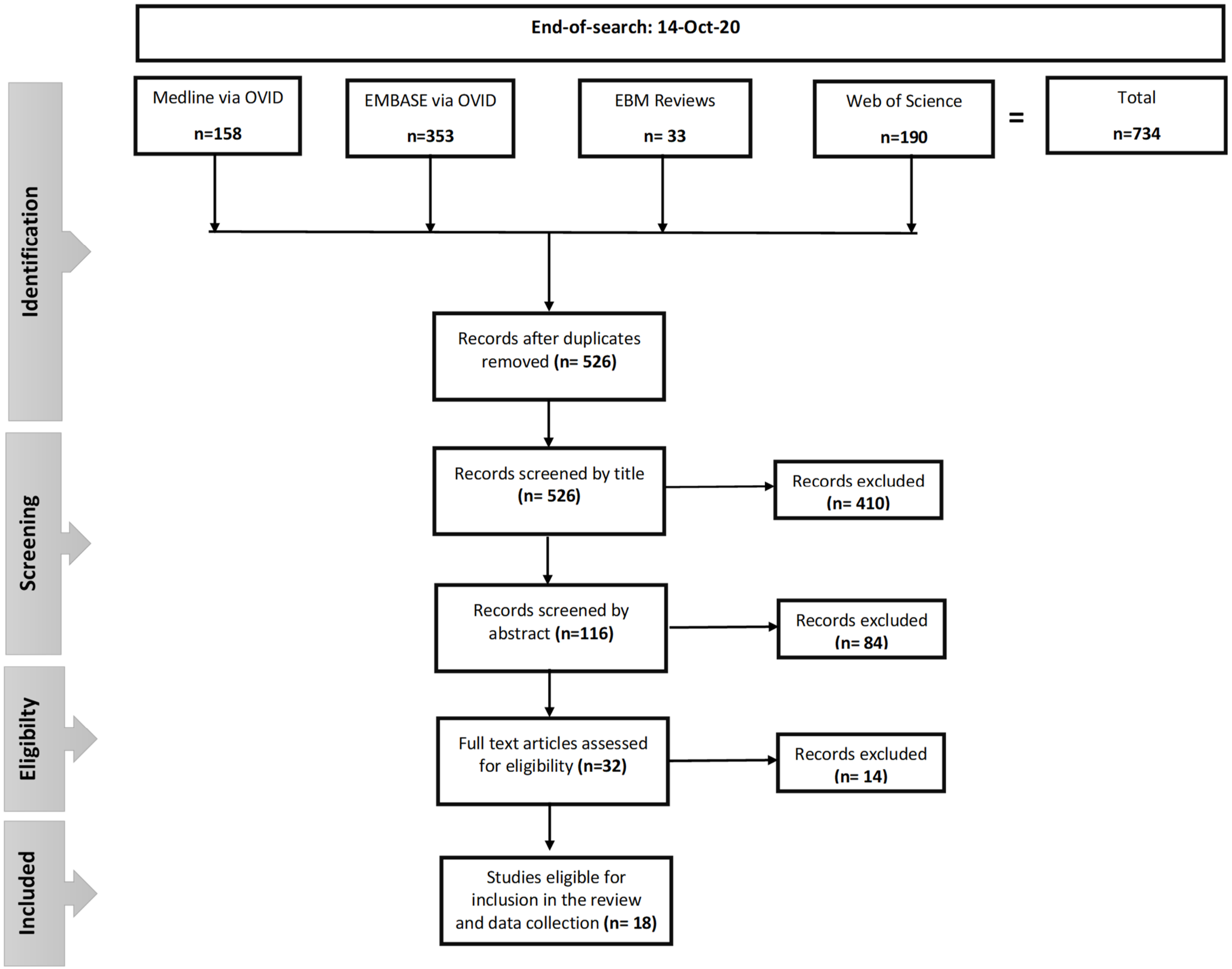

Supplementary Table S1: Key findings of miRNAs investigated singularly.

miRNAs	Author, Direction of dysregulation reported	Fold change OLK/OED vs N	Fold change OLK/OED vs OSCC	Fold change N vs OSCC	Fold change progressive OLK/OED vs Non progressive OLK/OED
**miR 181c**	**(Yang *et al.*, 2013) ↓**	**NS**	**NS**	**NS**	**↓15.40 (p<0.011)**
**miR 211**	**Check again for new data (excel sheet dated 7/2/21)**				
**miR 204-5p**	**(Philipone *et al.*, 2016) ↑↓**	**NS**	**NS**	**NS**	**Progressive Vs non-progressive: 1.60 (p<0.250).**<br>**↓ in deep sequencing analysis and ↑ in progressive group in the validation set (Nss)**
**miR 222-3p**	**(Chang *et al.*, 2018) ↓**	**↓ (p<0.0001)**	**NS**	**↓ (p<0.0001)**	**NS**
**miR 423-5p**	**(Chang *et al.*, 2018) ↑**	**NS**	**↑in OSCC (p<0.001)**	**↑(p<0.001)**	**NS**
**miR 150-5p**	**(Chang *et al.*, 2018) ↑**	**NS**	**↑in OSCC (p<0.001)**	**↑(p<0.001)**	**NS**
**miR-133b**	**(Cervigne *et al.*, 2009) ↓**	**↓ 0.013 Nss (p=1)**	**NS**	**↓0.319 Nss (p=1)**	**NS**
**miR 494**	**(Harrandah *et al.*, 2016) ↓**	**NS**	**↓ (0.015)**	**NS**	**NSD**
**miR 375**	**(Harrandah *et al.*, 2016) ↓**	**NS**	**↓ (P=0.0001)**	**NS**	**↓ 8 (P=0.0004)**
**miR 208b-3p**	**(Philipone *et al.*, 2016) ↑**	**NS**	**NS**	**NS**	**Progressive Vs**<br>**Non-progressive: ↑1.73 (p<0.049)**
**miR 129-2-3p**	**(Philipone *et al.*, 2016) ↓↑**	**NS**	**NS**	**NS**	**↓ in deep sequencing analysis and ↑ in validation set (Nss).**<br>**Progressive Vs non-progressive: ↑1.36 (p<0.391)**
**miR 3065-5p**	**(Philipone *et al.*, 2016) ↓**	**NS**	**NS**	**NS**	**↓0.86 in progressive group. (p<0.564)**

**miR 24**	**(Prasad *et al.*, 2017) ↓**	**CNRQ ratios: HNE/dysplasia ↓3.74 (p<0.05)**	**NS**	**CNRQ ratios: HNE/OSCC↓ 10.63 (P<0.05)**	**NS**
**miR 26b**	**(Prasad *et al.*, 2017) ↓**	**CNRQ ratios: HNE/OED ↓ 7.23 (p<0.05)**	**CNRQ ratios: OSCC/OED ↓ 2.77 (P<0.05)**	**CNRQ ratios: HNE/OSCC ↓ 2.58 (P<0.05)**	**NS**
**miR 155**	**(Prasad *et al.*, 2017) ↓**	**CNRQ ratios: HNE/OED ↓ 26.65 (P<0.05)**	**NS**	**CNRQ ratios: HNE/OSCC ↓ 8.67 (P<0.05)**	**NS**
**miR 127**	**(Prasad *et al.*, 2017) ↑**	**CNRQ ratios: OED/HNE: ↑ 14.87 (P<0.05)**	**CNRQ ratios: OED/OSCC: ↑ 8.95 (P<0.05)**	**NS**	**NS**
**miR 197**	**(Prasad *et al.*, 2017) ↑**	**NS**	**NS**	**NS**	**NS**
**miR 197-3p**	**(Yang *et al.* 2013) ↓**	**NS**	**NS**	**NS**	**↓16.89 (p<0.042)**
**miR 210**	**(Prasad *et al.*, 2017) Unchanged**	**CNRQ Ratio – Unchanged (p>0.05)**	**NS**	**CNRQ Ratio – Unchanged (p>0.05)**	**NS**
**miR 19b**	**(Prasad *et al.*, 2017) Unchanged**	**CNRQ Ratio – Unchanged (p>0.05)**	**NS**	**CNRQ Ratio – Unchanged (p>0.05)**	**NS**
**miR 19a-3p**	**(Yang *et al.*, 2013) ↑**	**NS**	**NS**	**NS**	**↑9.24 (p<0.007)**
**miR 205**	**(Prasad *et al.*, 2017) Unchanged**	**CNRQ Ratio – Unchanged (p>0.05)**	**NS**	**CNRQ Ratio – Unchanged (p>0.05)**	**NS**
**miR 145**	**(Zahran *et al.*, 2015) ↓**	**With OED: ↓ (P<0.001)**<br>**Without OED: ↓ (P<0.001)**	**With OED:0.6 (nss)**<br>**Without OED: Nss**	**↓ (p<0.001)**	**NS**
**miR 145-5p**	**(Yang *et al.*, 2013) ↓**	**NS**	**NS**	**NS**	**↓12.75 (p<0.01)**
**miR 125a**	**(Santhi *et al.*, 2013) ↓**	**↓ 0.39 (mean qrt exp) (p<0.0001)**	**NS**	**↓0.23 (P<0.0001)**	**NS**
**miR 125b-5p**	**(Yang *et al.*, 2013) ↓**	**NS**	**NS**	**NS**	**↓6.80 (p<0.01)**

**miR 16**	**(Santhi *et al.*, 2013) ↓**	**↓0.6 (p<0.0001)**	**NS**	**↓0.38 (P<0.0001)**	**NS**
**miR 96**	**(Santhi *et al.*, 2013) ↑**	**↑5.42 (p<0.0001)**	**NS**	**↑78.64 (P<0.0001)**	**NS**
**miR 129-5p**	**(Chen *et al.*, 2018) ↓**	**NSD**	**OLK-OSCC vs OLK : ↓ (p=0.0041)**	**OLK-OSCC vs N : ↓ (p=0.0001)**	**NS**
**miR 296-5p**	**(Chen *et al.*, 2018) ↓**	**NSD**	**OLK-OSCC vs OLK : ↓ (p=0.0014)**	**OLK-OSCC vs N : ↓ (p=0.0002)**	**NS**
**miR 450b-5p**	**(Chen *et al.*, 2018) ↑**	**Normal vs OLK: ↓ (p=0.046)**	**OLK-OSCC vs OLK : ↑ (p=0.0007)**	**OLK-OSCC vs N: ↑ (p=0.0001)**	**NS**
**miR196b**	**(Lu *et al.*, 2015) ↑**	**↑14.8 (p<0.01)<br><br>OR 46.2 (P<0.0001)**	**NS**	**↑17.0 (p<0.0001)<br><br>OR 189 (P<0.0001)**	**NS**
**miR 146b**	**(Cervigne *et al.*, 2009) ↑**	**NS**	**NS**	**NS**	**↑**
**miR 372**	**(Kao *et al.*, 2015) ↑↓**	**↑**	**NS**	**↓**	**NS**
**let 7i**	**(Kao *et al.*, 2015) ↑↓**	**↓**	**NS**	**↑**	**NS**
**let 7a-5p**	**(Yang *et al.*, 2013) ↓**	**NS**	**NS**	**NS**	**↓5.74 (p<0.039)**
**miR 10b-5p**	**(Yang *et al.*, 2013) ↑**	**NS**	**NS**	**NS**	**↑ 11.51 (p<0.008)**
**miR 99a-5p**	**(Yang *et al.*, 2013) ↓**	**NS**	**NS**	**NS**	**↓ 6.8 (p<0.011)**
**miR 99b-5p**	**(Yang *et al.*, 2013) ↓**	**NS**	**NS**	**NS**	**↓18.75(p<0.003)**
**miR 100-5p**	**(Yang *et al.*, 2013) ↓**	**NS**	**NS**	**NS**	**↓8.33 (p<0.01)**
**miR 331-3p**	**(Yang *et al.*, 2013) ↓**	**NS**	**NS**	**NS**	**↓6.84 (p<0.01)**
**miR 15a-5p**	**(Yang *et al.*, 2013) ↓**	**NS**	**NS**	**NS**	**↓12.51 (p<0.002)**
**miR 708**	**(Yang *et al.*, 2013) ↑**	**NS**	**NS**	**NS**	**↑11.89 (p<0.014)**
**miR 150-5p**	**(Yang *et al.*, 2013) ↓**	**NS**	**NS**	**NS**	**↓5.63 (p<0.002)**
**miR 30e-3p**	**(Yang *et al.*, 2013) ↑**	**NS**	**NS**	**NS**	**↑20.41 (p<0.002)**
**miR 30a-3p**	**(Yang *et al.*, 2013) ↑**	**NS**	**NS**	**NS**	**↑3.80 (p<0.049)**
**miR 335-5p**	**(Yang *et al.*, 2013) ↑**	**NS**	**NS**	**NS**	**↑2.08 (p<0.039)**
**miR 144***	**(Yang *et al.*, 2013) ↑**	**NS**	**NS**	**NS**	**↑12.22 (p<0.005)**
**miR 25-3p**	**(Yang *et al.*, 2013) ↑**	**NS**	**NS**	**NS**	**↑12.88 (p<0.017)**
**miR 660-5p**	**(Yang *et al.*, 2013) ↑**	**NS**	**NS**	**NS**	**↑27.92 (p<0.02)**
**miR 140-5p**	**(Yang *et al.*, 2013) ↑**	**NS**	**NS**	**NS**	**↑16.23 (p<0.012)**
**miR 590-5p**	**(Yang *et al.*, 2013) ↑**	**NS**	**NS**	**NS**	**↑15.79 (p<0.004)**

**miR-17-5p**	**(Cervigne *et al.*, 2009) ↓**	**↓0.351 Nss (p=0.98)**	**NS**	**↓0.644 Nss (p=0.97)**	**NS**
**miR-106b**	**(Cervigne *et al.*, 2009) ↑**	**↑ 1.157 (p=0.067)**	**NS**	**↑1.134 (P=0.054)**	**NS**
**miR-518b**	**(Cervigne *et al.*, 2009) ↑**	**↑2.557 (P<0.01)**	**NS**	**↑3.076 (p<0.01)**	**↑ in progressive**
**miR-520g**	**(Cervigne *et al.*, 2009) ↑**	**↑2.57 (P<0.01)**	**NS**	**↑9.932 (P<0.01)**	**↑ in progressive**
**miR-649**	**(Cervigne *et al.*, 2009) ↑**	**↑3.77 (P<0.01)**	**NS**	**↑3.464 (P<0.01)**	**↑ in progressive**

OSCC: Oral Squamous Cell Carcinoma; N: normal mucosa; OLK: Oral Leukoplakia; OED: Oral Epithelial Dysplaisa; NS- not specified; NSS- not statistically significant;

OPMD: Oral Potentially Malignant Disorders; NSD: No significant difference.
